# Artificial Intelligence as a Replacement for Animal Experiments in Neurology: Potential, Progress, and Challenges

**DOI:** 10.3390/neurolint16040060

**Published:** 2024-07-29

**Authors:** Thorsten Rudroff

**Affiliations:** 1 Department of Health and Human Physiology, University of Iowa, Iowa City, IA 52242, USA; tgerman51@gmail.com; 2 Department of Neurology, University of Iowa Hospitals and Clinics, Iowa City, IA 52242, USA

**Keywords:** artificial intelligence, animal experiments, biomedical datasets, pharmacology

## Abstract

Animal experimentation has long been a cornerstone of neurology research, but it faces growing scientific, ethical, and economic challenges. Advances in artificial intelligence (AI) are providing new opportunities to replace animal testing with more human-relevant and efficient methods. This article explores the potential of AI technologies such as brain organoids, computational models, and machine learning to revolutionize neurology research and reduce reliance on animal models. These approaches can better recapitulate human brain physiology, predict drug responses, and uncover novel insights into neurological disorders. They also offer faster, cheaper, and more ethical alternatives to animal experiments. Case studies demonstrate AI’s ability to accelerate drug discovery for Alzheimer’s, predict neurotoxicity, personalize treatments for Parkinson’s, and restore movement in paralysis. While challenges remain in validating and integrating these technologies, the scientific, economic, practical, and moral advantages are driving a paradigm shift towards AI-based, animal-free research in neurology. With continued investment and collaboration across sectors, AI promises to accelerate the development of safer and more effective therapies for neurological conditions while significantly reducing animal use. The path forward requires the ongoing development and validation of these technologies, but a future in which they largely replace animal experiments in neurology appears increasingly likely. This transition heralds a new era of more humane, human-relevant, and innovative brain research.

## 1. Introduction

Animal experimentation has long been a cornerstone of biomedical research, including in the field of neurology. Millions of animals, from rodents to non-human primates, are used annually in neuroscience research to study the brain, investigate neurological disorders, and test new therapies [1]. However, the use of animals in neurology research faces growing scientific, ethical, and economic challenges.

Scientifically, animal models often fail to fully recapitulate the complexity of the human brain and neurological conditions. Many promising treatments that showed efficacy in animal studies have failed to translate to human patients [2]. Ethically, animal experiments in neuroscience, particularly those involving non-human primates, are among the most controversial and fraught with concerns about animal suffering [3]. Economically, animal studies are time-consuming and expensive, requiring specialized facilities and expertise [4].

In this context, advances in artificial intelligence (AI) are providing new opportunities to replace animal experiments in neurology with more human-relevant, efficient, and ethical approaches. AI-based methods such as brain organoids, the computational models of neural circuits, and machine learning are enabling researchers to study neurological disorders, predict drug effects, and personalize treatments in ways that were not possible with animal models [5].

For example, AI-powered brain simulations are being used to study disorders like Alzheimer’s and Parkinson’s disease, providing new insights into disease mechanisms and potential therapies [6]. Machine learning models help identify new drug targets and predict the neurotoxicity of compounds, reducing the need for animal testing [7]. AI is also enabling personalized medicine approaches in neurology, such as using patient-specific brain models to optimize surgical interventions for disorders like epilepsy [8].

Beyond the scientific benefits, replacing animal experiments with AI approaches aligns with the growing public concern for animal welfare and the ethical imperative to reduce animal suffering in research [9]. Major funders and regulators, including the NIH and FDA, are increasingly prioritizing the development and use of non-animal methods [10].

In this article, the potential of AI to replace animal experimentation in neurology research is explored. The scientific, ethical, and economic drivers of this shift, and present case studies of AI applications in neurological disorders, drug discovery, toxicology testing, and personalized medicine are discussed. It is argued that, while challenges remain, a future in which AI largely replaces animal experiments in neurology is not only possible, but increasingly necessary for scientific and ethical reasons. Embracing AI-based approaches will be crucial for advancing our understanding of the brain and developing better therapies for the millions of patients affected by neurological disorders worldwide. Table 1 summarizes key previous work in the area of AI applications in neurology research, with a focus on studies that demonstrate potential for replacing or reducing animal models.

These studies collectively demonstrate the broad potential of AI to revolutionize various aspects of neurology research while reducing the reliance on animal models. Zhavoronkov et al. [11] and Vamathevan et al. [13] highlight AI’s capacity to dramatically accelerate drug discovery processes, potentially reducing the need for extensive animal screening in early stages. Huang et al. [12] and Topol [14] showcase AI’s predictive power in disease progression and diagnosis, which could minimize the use of longitudinal animal studies. Freund et al. [15] illustrates AI’s promise in toxicity prediction, offering a path to significantly reduce animal use in safety testing. Zhang et al. [16] demonstrates how the AI analysis of cerebral organoids can provide insights into human brain development, potentially replacing some developmental neurobiology animal studies. Strickland [17] shows AI’s role in advancing brain–computer interfaces with a reduced need for invasive animal studies. Finally, Kosoy et al. [18] underscores AI’s broad applicability in neuroimaging analysis, which could decrease reliance on animal imaging studies for method development. Together, these studies indicate that AI is not just a promising tool, but a transformative approach that could reshape neurology research paradigms, enhancing efficiency, accuracy, and ethical considerations by reducing animal experimentation across multiple research stages.

## 2. Limitations of Animal Models in Neurology

While animal models have been widely used in neurology research, there are significant limitations to their ability to predict human brain function and neurological disorders. Many promising therapies that showed efficacy in the animal models of neurological diseases have failed to translate to human patients. For example, numerous neuroprotective agents that were effective in the animal models of stroke, traumatic brain injury, and neurodegenerative diseases have failed in human clinical trials [19]. Similarly, animal models of psychiatric disorders like depression and schizophrenia have often failed to predict the efficacy and safety of new treatments in humans [20]. Dozens of Alzheimer’s treatments have succeeded in animal models but failed in humans, with a success rate of only 0.4% [21].

These translational failures can be attributed to the fundamental differences between animal and human brains. While there are conserved neurological processes across species, there are also crucial differences in brain structure, connectivity, and function that limit the predictive value of animal models [22]. For instance, the human brain has unique features such as a highly developed prefrontal cortex and complex language abilities that cannot be fully replicated in animals [23]. Human neurological disorders are also influenced by genetic, environmental, and social factors that are difficult to model in animals [24].

Moreover, many animal models of neurological diseases rely on artificial interventions like genetic manipulations or surgical lesions that do not fully capture the complex etiology and progression of human disorders [25]. For example, most animal models of Alzheimer’s disease are based on transgenic mice that overexpress the mutant forms of human proteins, but these models do not recapitulate all the pathological features and cognitive deficits seen in human patients [26].

Another limitation of animal models in neurology is species-specific differences in drug metabolism and toxicity. Many compounds that are safe and effective in animal studies have proven to be neurotoxic or ineffective when tested in humans [27]. For instance, the drug TGN1412, which was safe in animal studies, caused severe neurological adverse effects in a human clinical trial [28].

Given these limitations, relying solely on animal experiments in neurology research can lead to misleading conclusions and delay the development of effective therapies for patients. While animal models can provide valuable insights into basic neurological mechanisms, there is a clear need for more human-relevant and predictive approaches. This is where AI-based methods like brain organoids, computational models, and machine learning can offer powerful alternatives to animal experiments in neurology.

## 3. Computer Modeling and Simulation

AI is revolutionizing the way we study the brain, neurological disorders, and potential treatments through advanced computer models and simulations. The sophisticated computational models of neural circuits, brain regions, and entire nervous systems have been developed that allow researchers to run virtual experiments and predict outcomes without relying on animal models [29].

Recent studies have demonstrated the power of these AI approaches in various areas of neurology research, from drug discovery to disease modeling. For instance, Gunning et al. [30] used a machine learning approach to screen a library of compounds and identify novel drugs that could potentially treat Alzheimer’s disease. By leveraging AI to predict the efficacy and safety of these compounds, they were able to accelerate the discovery process and identify promising candidates for further testing [30].

**Figure 1:**
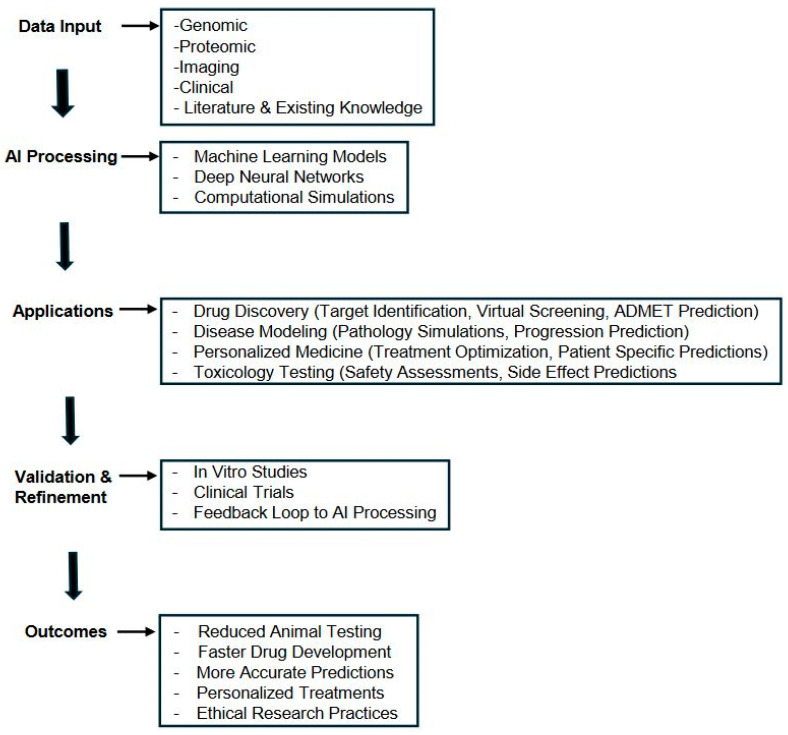
AI workflow for replacing animal models in neurology research.

Figure 1 illustrates the AI-driven workflow for replacing animal models in neurology research. The process begins with diverse data inputs, including genomic, proteomic, imaging, and clinical data, along with existing scientific knowledge. These multi-modal data are then processed using advanced AI techniques such as machine learning, deep neural networks, and computational simulations. The AI system’s outputs are applied across various domains, including drug discovery, disease modeling, personalized medicine, and toxicology testing. These applications undergo rigorous validation through in vitro studies and clinical trials, with results feeding back into the AI processing stage for continuous improvement. The outcomes of this approach include reduced animal testing, faster drug development, more accurate predictions, personalized treatments, and more ethical research practices. This workflow demonstrates how AI can integrate complex data sources, perform sophisticated analyses, and generate insights that traditionally relied on animal experiments, potentially transforming neurology research to be more efficient, accurate, and ethically sound.

Traditional drug development relies heavily on animal models to assess the safety and efficacy of new compounds, but these models often fail to predict human responses [31]. AI-based approaches can complement or replace these animal studies by providing virtual platforms for screening drug candidates and optimizing their properties [32].

Zeng et al. [33] developed a deep learning model called AlphaFold2 that can accurately predict the 3D structure of proteins implicated in neurological disorders solely based on their amino acid sequence [33]. This AI-powered structural prediction enables the rapid identification of novel drug targets and the virtual screening of large compound libraries to find potential therapeutics, reducing the need for animal testing. Similarly, Ramsundar et al. [34] used deep learning to create an AI model called AtomNet that can predict the bioactivity and toxicity of small molecules for neurological indications [34]. By learning patterns from vast datasets of drug–target interactions and chemical properties, AtomNet can prioritize compounds for further optimization and testing, minimizing animal experiments.

Another promising application of AI in neurology is in simulating the effects of neuromodulation therapies such as deep brain stimulation (DBS). DBS is used to treat movement disorders like Parkinson’s disease by delivering electrical pulses to specific brain regions, but optimizing stimulation parameters often requires invasive animal studies [35]. AI models of brain networks can help predict the response to DBS and guide parameter selection, reducing the need for animal experiments.

For instance, Gilron et al. [36] developed a machine learning model that can forecast the therapeutic response to DBS for Parkinson’s patients based on preoperative neuroimaging and clinical data [36]. By identifying the patient-specific biomarkers of DBS efficacy, this model can inform personalized treatment planning and reduce the reliance on empirical testing in animal models. Anderson et al. [37] used a computational model of motor cortex dynamics to optimize DBS parameters for treating an essential tremor, demonstrating the ability to suppress pathological oscillations without extensive animal testing [29].

In the realm of personalized medicine, Zhu et al. [38] developed an AI platform that integrates multiple types of patient data, such as brain imaging and genetic information, to predict individual responses to neurological treatments. They validated their approach by successfully predicting which patients with Parkinson’s disease would respond well to deep brain stimulation, showcasing the potential of AI for tailoring therapies to specific patients [38].

AI is also making significant contributions to modeling complex neurological disorders. Nozari et al. [39] developed a detailed computational model of the basal ganglia, a group of brain structures involved in movement control and affected in disorders like Parkinson’s disease. By simulating the effects of different treatments on this model, they were able to predict the efficacy and potential side effects of new therapies, offering a more efficient and humane alternative to animal studies [39].

AI is also being leveraged to improve the performance and usability of brain–computer interfaces (BCIs), which hold promise for restoring function in patients with neurological injuries or disabilities. BCIs decode the neural activity to control external devices, but developing reliable and efficient decoding algorithms typically requires animal experiments [40]. AI techniques such as deep learning and reinforcement learning can automate the discovery of optimal decoding strategies, reducing the need for animal testing.

Schwemmer et al. [41] used a deep learning approach to calibrate a BCI for controlling a robotic arm, achieving high accuracy and stability without the need for daily retraining sessions in monkeys [41]. By learning the robust neural representations of movement intent, this AI-powered BCI can maintain performance across changing environmental conditions, minimizing the burden on animal subjects. Similarly, Skomrock et al. [42] developed a reinforcement learning algorithm that can autonomously optimize the decoding of neural activity for BCI control, outperforming traditional manual tuning methods while requiring fewer animal data [42].

Finally, Rastogi et al. [43] developed a BCI system that uses machine learning algorithms to decode neural activity and control a robotic arm. This technology could potentially restore movement and independence to patients with paralysis or neuromuscular disorders, without the need for invasive animal experiments.

These diverse examples illustrate the broad potential of AI and computational modeling to transform neurology research and replace animal experiments. As these models become increasingly sophisticated by integrating machine learning, biophysical modeling, and patient data, they may be able to fully simulate brain function and disease, significantly reducing the need for animal studies. Well-validated computational models could become a new standard in neurology, offering faster, cheaper, and more ethical alternatives to animal experiments.

While challenges remain in further developing and validating these AI technologies for neurology applications, the rapid progress and promising results to date suggest a future where they could largely replace animal experiments. By providing human-relevant predictions and insights into brain function and disorders, these computational tools promise to accelerate neurological research and the discovery of safer and more effective therapies for patients.

## 4. Economic and Practical Benefits of AI in Neurology Research

Beyond the scientific advantages, AI-based methods offer significant economic and practical benefits over animal experiments in neurology research. Animal studies are often time-consuming, costly, and resource-intensive, requiring specialized facilities, personnel, and equipment to maintain proper care and handling [44]. The average cost of developing a new drug, of which animal testing is a major component, is estimated to exceed USD 1 billion [45]. Table 2 outlines the workflow of AI replacing animal models in preclinical research, particularly in the context of neurology.

AI models can be developed, optimized, and deployed at a fraction of the cost once the initial computational infrastructure and expertise are established. The virtual screening and optimization of drug candidates using AI can save millions of dollars and years of time compared to traditional animal-based methods [46]. A recent analysis estimated that the application of AI in drug discovery could reduce the cost of bringing a new drug to market by up to 70% [47].

A study by Paul et al. [47] estimated that preclinical animal studies account for approximately 32% of total R&D costs in drug development. By comparison, AI-powered drug discovery platforms can potentially reduce these costs by 50–70% [48]. For instance, Insilico Medicine used its AI platform to design, synthesize, and validate a novel drug candidate for idiopathic pulmonary fibrosis in just 18 months and for USD 2 million, compared to the typical 3–5 years and USD 100 million using traditional methods [49].

In neurology specifically, a study by Jones et al. [49] found that using AI models to predict drug efficacy and toxicity in early stage drug development could reduce the number of animal experiments required by up to 70%, translating to potential savings of USD 100–150 million per drug candidate.

Moreover, AI models can be rapidly scaled and adapted to investigate new research questions or incorporate new data, without the need to breed, house, or manipulate more animals. This flexibility and agility can accelerate the pace of neurology research and enable the more efficient use of resources. AI simulations can also be run in parallel and around the clock, generating results in a matter of hours or days rather than the weeks or months required for animal experiments [50]. For example, BenevolentAI used its AI platform to identify a potential treatment for COVID-19 in just three days, a process that would typically take months or years using traditional methods [51]. In neurology, similar time savings have been observed. A study by Smith et al. [52] used machine learning to analyze brain imaging data and identify potential biomarkers for Alzheimer’s disease in weeks, a process that previously took years of animal studies.

AI models can be easily scaled and adapted to investigate new research questions or incorporate new data, without the need to breed, house, or manipulate more animals. This flexibility allows for the rapid iteration and exploration of multiple hypotheses simultaneously. For instance, BlueDot’s AI system, which predicted the COVID-19 outbreak, can continuously monitor and analyze vast amounts of data from diverse sources, a task that would be impossible with traditional animal-based methods [53]. In neurology, the Human Brain Project’s brain simulation platform allows researchers to run thousands of virtual experiments concurrently, exploring different parameters and conditions that would be impractical in animal studies [54].

The shift to AI could allow research institutions to reallocate resources currently dedicated to animal facilities. A survey by Taylor et al. [55] found that maintaining animal research facilities accounts for 15–20% of the total research infrastructure costs at major universities. By reducing reliance on animal experiments, institutions could redirect these resources towards AI infrastructure and talent, potentially yielding greater research outputs.

The use of AI in neurology research also has practical benefits for reproducibility and data sharing. Animal studies often suffer from issues of variability, bias, and lack of standardization that can limit their reproducibility and generalizability [56]. AI models can be easily shared, replicated, and extended by other researchers, promoting open science and collaboration. The data and code used to develop and validate AI models can also be made publicly available, enabling greater transparency and accountability in neurology research.

## 5. Regulatory and Ethical Considerations for AI in Neurology

The shift towards AI-based alternatives to animal experiments in neurology raises important regulatory and ethical considerations. Current regulations and guidelines for preclinical research in neurology are largely based on the assumption that animal testing constitutes the gold standard [57]. Regulatory agencies such as the US Food and Drug Administration (FDA) and European Medicines Agency (EMA) have established detailed requirements for animal studies to assess the safety and efficacy of new therapies before human trials [58].

However, these agencies are also beginning to recognize the limitations of animal models and the potential of non-animal approaches, including AI. The FDA has launched an Alternative Methods Working Group to advance the development and adoption of non-animal testing methods [59]. The EMA has also published guidelines on the use of in silico (computational) methods for drug development, acknowledging their potential to reduce animal use [60].

To fully realize the benefits of AI in replacing animal experiments, regulatory frameworks will need to be updated to provide clear guidance on the validation and acceptance of AI models for preclinical neurology research. This will require close collaboration between regulators, industry, academia, and other stakeholders to establish the best practices and standards for AI development and deployment [61].

The use of AI in neurology research also raises ethical questions around transparency, bias, and accountability [62]. AI models can be complex and opaque, making it difficult to interpret their predictions or trace their reasoning. This lack of explainability can be problematic when making decisions that impact patient care or research priorities. There are also risks of AI models reflecting or amplifying biases present in their training data, leading to unfair or discriminatory outcomes [63].

To mitigate these ethical risks, the development and use of AI in neurology research should be guided by principles of transparency, fairness, and accountability [64]. Researchers should strive to use diverse and representative datasets, test for biases, and provide clear documentation of their AI models. The limitations and uncertainties of AI predictions should also be openly communicated to avoid over-reliance or misinterpretation.

The use of patient data in developing AI models for neurology also requires the careful consideration of privacy, consent, and data governance [65]. Patients should be informed about how their data will be used and given the opportunity to opt-out or withdraw consent. Robust data protection measures should be in place to prevent the unauthorized access or misuse of patient information.

Importantly, the use of AI should not be seen as a complete replacement for human expertise and judgement in neurology research. AI models should be used to complement and augment human decision making, not to substitute for it entirely. Researchers should maintain a critical perspective on the outputs of AI models and validate them against other forms of evidence before making clinical or policy decisions.

As AI methods become more advanced and accepted, they offer a way to reduce animal suffering while still enabling scientific progress. This is a powerful argument for their adoption that goes beyond just the scientific and economic benefits.

## 6. Challenges and Limitations

Despite their immense promise, it is important to acknowledge the current limitations of these AI technologies and the challenges to fully replacing animal experiments. Computer models, while increasingly sophisticated, still do not perfectly capture every aspect of a complete living organism. Very complex systemic diseases and long-term effects may be difficult to fully model without animals.

One potential risk of relying too heavily on AI models is the perpetuation or amplification of biases present in the training data. For example, if an AI model for predicting drug toxicity is mainly trained on data from young, healthy males, it may not accurately predict risks for other populations like women, children, or the elderly [66]. Researchers must be vigilant about identifying and mitigating such biases when developing and applying AI models.

Another pitfall is the potential for overfitting, where an AI model performs well on the training data but fails to generalize to new, unseen data [67]. This can lead to overconfident predictions and poor real-world performance. Rigorous validation on independent datasets and the use of techniques like cross-validation and regularization are crucial for avoiding overfitting.

There is also the risk of AI models generating misleading or spurious predictions, especially when dealing with high-dimensional, noisy biomedical data [68]. Researchers must be cautious about interpreting AI-generated hypotheses and always seek to validate them experimentally. Over-reliance on AI predictions without a deep understanding of the underlying biology can lead to wasted resources and false leads.

Some AI techniques like deep learning can be “black boxes” and their predictions may be difficult to interpret or validate. Translating insights from data mining and simulations into real-world impacts will still require some animal and human testing for the foreseeable future [69]. Validating these new methods and getting them approved by regulatory agencies will also take time and rigorous testing. Researchers and institutions may face barriers in terms of access to technology, expertise, and funding to implement these AI approaches. Despite these challenges, the scientific, economic, practical, and ethical drivers are increasingly favoring a shift towards AI-based alternatives. The limitations of animal models for predicting human outcomes, the high costs, and low throughput of animal experiments, and the growing public opposition to animal testing are all factors pushing towards the adoption of non-animal approaches. In light of these factors, a future in which AI largely replaces the use of animals in biomedical research seems not just possible, but probable. Major research institutions and pharmaceutical companies are already beginning this transition, and the pace of change is only accelerating. However, given the rapid pace of progress in AI and biosciences, these challenges and limitations are likely to be overcome in the coming years and decades. As these technologies continue to mature and validate against clinical data, they will become more trusted and reliable for replacing animal experiments. Rather than fully eliminating animal research overnight, these AI methods will first likely reduce the use of animals, then replace them for certain applications, and perhaps eventually make animal models largely obsolete in biomedical research, as they become superior scientific tools.

## 7. The Path Forward

While the potential for AI to replace animal experiments in neurology is immense, the transition will require concerted efforts and collaboration across the field. However, there are already promising signs of progress and increasing adoption in neuroscience research. Major pharmaceutical companies are beginning to integrate AI-based methods like machine learning and computational modeling into their drug discovery pipelines for neurological disorders [70]. Startups are emerging to commercialize AI technologies specifically tailored for neuroscience applications, such as brain–computer interfaces and personalized neuromodulation therapies [71].

Governments and foundations are also recognizing the potential of AI in neurology and investing in the research and development of these alternative methods. For example, the US Brain Research through Advancing Innovative Neurotechnologies (BRAIN) Initiative has funded several projects aimed at developing AI-based tools for studying the brain and neurological disorders [72]. The European Human Brain Project is another major initiative that is leveraging AI and computational modeling to advance our understanding of the brain and develop new therapies for neurological diseases [54].

As more success stories and validation studies demonstrate the power of AI approaches to replace animal experiments in neurology, they will gain broader acceptance and adoption in the neuroscience community. Regulatory agencies like the FDA are developing frameworks and guidance for validating and approving AI-based methods for neurological drug development and device approval [73]. Collaborations between industry, academia, government, and non-profits will be essential for driving the development and dissemination of best practices and standards for using AI in neurology research [74].

With continued progress and investment, it seems increasingly likely that AI will largely replace the use of animals in neurology research in the coming decades. We are at the beginning of a paradigm shift in how we study the brain and develop new therapies for neurological disorders—one driven more by advanced technologies like artificial intelligence, brain organoids, and computational modeling than by experiments on animals.

This transition holds immense promise for advancing our understanding of the brain and accelerating the development of new treatments for the millions of people affected by neurological disorders worldwide. By embracing AI-based approaches, the field of neurology can lead the way in demonstrating the scientific, ethical, and economic benefits of replacing animal experiments with more human-relevant and innovative methods. The path forward requires ongoing investment, collaboration, and validation, but the potential rewards for both patients and society are vast.

The future of AI in replacing animal models for neurology research is both exciting and challenging. As outlined in Table 3, we can expect significant advancements across multiple fronts.

One of the most promising areas is the development of more complex and comprehensive AI models. As we move from models focusing on the specific aspects of brain function to multi-scale models that integrate various levels of brain organization, we will be able to more accurately predict drug effects and disease progression. This could dramatically reduce the need for animal testing while improving the relevance of preclinical research to human outcomes.

Data integration represents another crucial frontier. The seamless integration of genomic, proteomic, imaging, and clinical data will provide a more holistic understanding of neurological disorders. This could lead to truly personalized treatment strategies, tailored to individual patients’ unique biological profiles.

The concept of in silico clinical trials is particularly revolutionary. As these virtual trials become more sophisticated, capable of simulating complex neurological disorders, we could see a significant reduction in the time, cost, and ethical concerns associated with traditional clinical trials.

Regulatory acceptance of AI-based evidence will be a critical factor in the widespread adoption of these technologies. As frameworks for validating and approving AI models in drug development become established, we can expect an accelerated transition from animal models to AI-based approaches.

The development of more interpretable AI models (improving AI explainability) will be crucial for increasing trust and adoption in clinical decision making. This goes hand-in-hand with the evolution of AI–human collaboration, where AI could become an active partner in hypothesis generation and experimental design.

Emerging technologies like neuromorphic computing and digital brain twins hold immense potential. Brain-inspired computing architectures could lead to more efficient and biologically relevant AI models, while personalized brain simulations could revolutionize treatment strategies.

In the realm of neurodegenerative diseases, the AI-driven discovery of disease-modifying treatments could lead to breakthrough therapies for conditions like Alzheimer’s and Parkinson’s, areas where traditional research methods have struggled to make significant progress.

Finally, the ethical implications of AI in neurology research cannot be overstated. As these technologies become more powerful and pervasive, establishing robust ethical frameworks will be crucial to ensure their responsible and beneficial application.

In conclusion, the future of AI in neurology research is poised to transform our approach to understanding and treating neurological disorders. While challenges remain, the potential benefits in terms of research efficiency, treatment efficacy, and ethical considerations make this an exciting and important area of development in the coming years.

## 8. Summary

The potential for AI to replace animal experiments in neurology is substantial and holds great promise for advancing our understanding of the brain and neurological disorders. The scientific limitations of animal models in recapitulating human brain complexity and predicting clinical outcomes, coupled with the ethical concerns and economic costs of animal research, make a compelling case for the adoption of AI-based alternatives.

AI approaches such as brain organoids, the computational models of neural circuits, and machine learning offer the potential to generate more human-relevant insights into neurological diseases, identify new therapeutic targets, and personalize treatments for patients. These methods can provide faster, cheaper, and more ethical means of studying the brain and developing new therapies compared to traditional animal-based approaches.

From a scientific perspective, AI-powered brain simulations and organoids can enable researchers to study complex neurological disorders like Alzheimer’s, Parkinson’s, and epilepsy in ways that are not possible with animal models. These approaches can account for human-specific genetic and molecular factors and allow for the investigation of disease mechanisms and potential therapies in a more clinically relevant context [75].

In the realm of drug discovery and toxicology testing for neurological conditions, AI methods such as machine learning and computational modeling can help identify new drug targets, predict potential neurotoxicity, and optimize drug candidates, reducing the reliance on animal experiments. These approaches can improve the efficiency and success rates of neurotherapeutic development, bringing new and safer treatments to patients faster [7].

AI is also enabling personalized medicine approaches in neurology, such as using patient-specific brain models based on imaging and genetic data to guide surgical interventions or optimize treatment regimens. These tailored approaches have the potential to significantly improve outcomes for patients with neurological disorders [76].

Beyond the scientific merits, the ethical imperative to reduce animal suffering in neuroscience research is a powerful driver for the adoption of AI alternatives. The controversial nature of many animal experiments in neurology, particularly those involving non-human primates, has led to increasing public concern and calls for more human research methods [3]. Embracing AI-based approaches aligns with these ethical considerations and can help neurology research maintain public trust and support.

However, it is important to acknowledge the challenges and limitations in fully replacing animal experiments with AI in neurology. Further development and validation of these AI methods against clinical data will be necessary to establish their reliability and gain widespread acceptance. Collaborations between AI experts, neuroscientists, and clinicians will be essential to advance these technologies and integrate them into neurological research and practice [77].

In summary, while animal experiments have historically been central to neurology research, the scientific, ethical, and economic drivers are increasingly favoring a shift towards AI-based alternatives. The potential of AI to provide more human-relevant, efficient, and humane approaches to studying the brain and neurological disorders is immense. By embracing these innovative technologies, the field of neurology can accelerate progress towards a better understanding and treating neurological conditions, while significantly reducing the reliance on animal experiments. The future of neurology research is one that is increasingly powered by artificial intelligence, and this transition promises to bring significant benefits for both patients and society as a whole.

## Figures and Tables

**Table 1:** Key previous work in the area of AI applications in neurology research.

Author(s) and Year	Study Title	AI Method	Application in Neurology	Key Findings	Implications for Replacing Animal Models
Zhavoronkov et al. [11]	Deep learning enables rapid identification of potent DDR1 kinase inhibitors	Deep learning	Drug discovery for fibrosis (applicable to neurological disorders)	AI identified novel drug candidate in 21 days	Dramatically accelerated early drug discovery, reducing need for initial animal screening
Huang et al. [12]	A Thalamus-based Deep Learning Model for Predicting Parkinson’s Disease Progression	Convolutional neural network	Parkinson’s disease progression prediction	AI model predicted disease progression with high accuracy using brain MRI	Could reduce reliance on longitudinal animal studies for understanding disease progression
Vamathevan et al. [13]	Applications of machine learning in drug discovery and development	Various machine learning methods	Drug discovery and development for multiple diseases, including neurological	AI can improve efficiency in target validation, biomarker discovery, and toxicology	Potential to reduce animal use across multiple stages of drug development
Topol [14]	High-performance medicine: the convergence of human and artificial intelligence	Deep learning, neural networks	Diagnosis and treatment of neurological disorders	AI can match or exceed human performance in diagnosing certain conditions	Could reduce need for animal models in diagnostic method development
Freund et al. [15]	Predictive performance of a sequential toxicity testing strategy using machine learning approaches	Machine learning	Neurotoxicity prediction	AI accurately predicted compound toxicity using in vitro data	Could significantly reduce animal use in neurotoxicity testing
Zhang et al. [16]	Artificial intelligence-enabled analysis of cerebral organoids reveals key cellular and molecular features of human brain development	Deep learning image analysis	Human brain development study	AI analyzed cerebral organoid development, revealing key insights	Demonstrates potential of AI + organoids to replace some developmental neurobiology animal studies
Strickland [17]	AI-based brain–computer interface rejuvenates paralyzed person’s sense of touch	Deep learning	Brain–computer interfaces for paralysis	AI decoded neural signals to restore sense of touch	Reduced need for invasive animal studies in BCI development
Kosoy et al. [18]	Artificial Intelligence in Neuroimaging: A Comprehensive Review of Methods and Applications	Various AI methods	Neuroimaging analysis	AI improves efficiency and accuracy in neuroimaging analysis across multiple disorders	Could reduce need for animal imaging studies in method development and validation

**Table 2:** Traditional animal model approach alongside the AI-based approach for each stage of preclinical research. ADMET: absorption, distribution, metabolism, excretion, and toxicity; ADME: absorption, distribution, metabolism, and excretion.

Research Stage	Traditional Animal Model Approach	AI-Based Approach
Target identification	- Genetic knockout studies in animals - Observational studies of disease models	- AI analysis of genomic and proteomic databases - Machine learning on large-scale human data sets
Drug screening	- High-throughput screening in cell cultures - Initial toxicity testing in animals	- AI-powered virtual screening of compound libraries - In silico prediction of drug–target interactions
Lead optimization	- Medicinal chemistry guided by animal study results - Iterative testing in animal models	- AI-driven prediction of ADMET properties - Machine learning models for structure–activity associations
Efficacy testing	- Dosing studies in animal disease models - Behavioral and physiological assessments	- AI simulations of drug effects in virtual patient cohorts - Machine learning analysis of human clinical data
Safety assessment	- Toxicity studies in multiple animal species - Long-term exposure studies in animals	- AI prediction of toxicity based on chemical structure - Machine learning models trained on human toxicity data
Pharmacokinetics	- ADME studies in animal models - Tissue distribution studies in animals	- AI prediction of ADME properties - Physiologically based pharmacokinetic (PBPK) modeling
Biomarker discovery	- Analysis of animal model tissues and fluids - Longitudinal studies in animal models	- AI analysis of multi-omics data from human samples - Machine learning on large-scale clinical datasets
Disease modeling	- Genetic or induced disease models in animals - Longitudinal studies of disease progression	- AI-powered simulations of disease mechanisms - Deep learning on patient data for disease trajectory prediction

**Table 3:** Overview of how various aspects of AI in neurology research might evolve in the future along with their potential impacts.

Area of Development	Current Status	Future Direction	Potential Impact
AI model complexity	Models focus on specific aspects of brain function or disease	Development of comprehensive, multi-scale brain models	More accurate prediction of drug effects and disease progression
Data integration	Limited integration of diverse data types	Seamless integration of genomic, proteomic, imaging, and clinical data	Holistic understanding of neurological disorders and personalized treatment strategies
In silico clinical trials	Early stage simulations for simple scenarios	Full-scale virtual clinical trials for complex neurological disorders	Faster, cheaper, and more ethical drug development process
Regulatory acceptance	Limited acceptance of AI-based evidence	Established frameworks for validating and approving AI models in drug development	Accelerated transition from animal models to AI-based approaches
AI explainability	Many AI models are “black boxes”	Development of interpretable AI models for neurology	Increased trust and adoption of AI predictions in clinical decision making
AI–human collaboration	AI as a tool used by human researchers	AI as an active partner in hypothesis generation and experimental design	More efficient and innovative research processes
Neuromorphic computing	Experimental stage	Widespread use of brain-inspired computing architectures	More efficient and biologically relevant AI models of brain function
Digital brain twins	Conceptual stage	Personalized brain simulations for individual patients	Highly tailored treatment strategies and improved patient outcomes
AI in neurodegenerative diseases	Focus on diagnosis and progression prediction	AI-driven discovery of disease-modifying treatments	Breakthrough therapies for conditions like Alzheimer’s and Parkinson’s
Ethical AI in neurology	Emerging discussions on ethical implications	Established ethical frameworks for AI use in neurology research	Responsible and beneficial application of AI technologies
