# Correction: Rudroff, T. Artificial Intelligence as a Replacement for Animal Experiments in Neurology: Potential, Progress, and Challenges. *Neurol. Int.* 2024, *16*, 805–820

**DOI:** 10.3390/neurolint18060106

**Published:** 2026-05-28

**Authors:** Thorsten Rudroff

**Affiliations:** 1Department of Health and Human Physiology, University of Iowa, Iowa City, IA 52242, USA; tgerman51@gmail.com; 2Department of Neurology, University of Iowa Hospitals and Clinics, Iowa City, IA 52242, USA

## Error in Table

In the original publication [1], there was a mistake in Table 1 as published. The table included incorrect references. The corrected Table 1 Key previous work in the area of AI applications in neurology research appears below.

Because the changes of the references citation in Table 1, some paragraphs refer to the relevant reference have also been changed as well (paragraph under Table 1, Sections 2 and 3, paragraphs below Figure 1, Section 4 references, Section 5 references, Section 6 references, Section 7 paragraph 1 altered text and references, summary references). 

## References

The reference list in the original publication contained incorrect citations due to a formatting and compilation error during manuscript preparation. With this correction, the order of some references has been adjusted accordingly. The author state that the scientific conclusions are unaffected. This correction was approved by the Academic Editor. The original publication has also been updated.

## Figures and Tables

**Table 1 neurolint-18-00106-t001:** Key previous work in the area of AI applications in neurology research.

Author(s) and Year	Study Title	AI Method	Application in Neurology	Implications for Replacing Animal Models
Ferreira & Carneiro [11]	AI-Driven Drug Discovery: A Comprehensive Review	Machine learning, deep learning	Drug discovery for neurological disorders	Dramatically accelerated early drug discovery, reducing need for initial animal screening
Shahid & Singh [12]	A deep learning approach for prediction of Parkinson’s disease progression	Deep learning	Parkinson’s disease progression prediction	Could reduce reliance on longitudinal animal studies for understanding disease progression
Petrella et al. [13]	Personalized Computational Causal Modeling of Alzheimer Disease Biomarker Cascade	Computational causal modeling	Alzheimer’s disease biomarker analysis	Enables patient-specific disease modeling without animal models
Ajisafe et al. [14]	The role of machine learning in predictive toxicology	Machine learning	Neurotoxicity prediction	Could significantly reduce animal use in neurotoxicity testing
Bai et al. [15]	AI-enabled organoids: Construction, analysis, and application	Deep learning image analysis	Organoid analysis for brain development	Demonstrates potential of AI with organoids to replace developmental neurobiology animal studies
Zhang et al. [16]	Modeling neurological disorders using brain organoids	Computational modeling	Disease modeling with organoids	Provides human-relevant disease models, reducing animal use
Ganzer et al. [17]	Restoring the Sense of Touch Using a Sensorimotor Demultiplexing Neural Interface	Deep learning	Brain–computer interfaces for paralysis	Reduced need for invasive animal studies in BCI development
Boutet et al. [18]	Predicting optimal deep brain stimulation parameters for Parkinson’s disease using functional MRI and machine learning	Adaptive algorithms	Personalized DBS for Parkinson’s disease	Enables patient-specific optimization, reducing animal testing
Lu et al. [19]	Toward personalized brain stimulation: Advances and challenges	Computational modeling	Personalized neuromodulation	Reduces reliance on animal models for treatment optimization
Monsour et al. [20]	Neuroimaging in the Era of Artificial Intelligence: Current Applications	Various AI methods	Neuroimaging analysis	Could reduce need for animal imaging studies in method development
Jumper et al. [21]	Highly accurate protein structure prediction with AlphaFold	Machine learning	Multimodal neuroimaging	Improves diagnostic accuracy with human data, reducing animal model dependency
Kalani & Anjankar [22]	Revolutionizing Neurology: The Role of AI in Advancing Diagnosis and Treatment	Various AI methods	Diagnosis and treatment in neurology	Demonstrates broad applicability of AI approaches, reducing animal experimentation
